# Prefrontal Cortical Near-Infrared Spectroscopy for Acute Pain Assessment in Infants: A Feasibility Study

**DOI:** 10.3390/jcm14072525

**Published:** 2025-04-07

**Authors:** Matthias Nissen, Ralf-Bodo Tröbs

**Affiliations:** 1 Department of Pediatric Surgery, Marien Hospital Witten, St. Elisabeth Gruppe, Ruhr-University of Bochum, Marienplatz, 58452 Witten, Germany; 2 Department of General-, Visceral-, and Pediatric Surgery, Section of Pediatric Surgery and Urology, St. Vinzenz Hospital, Academic Teaching Hospital of Georg-August-University Göttingen, Am Busdorf, 33908 Paderborn, Germany; pedsurgtroe@gmail.com

**Keywords:** prefrontal cortex, bilateral frontal near-infrared spectroscopy, NIRS, cerebral hemodynamics, nociceptive stimulus, pain assessment, surgical pain management, infants, pediatric surgery, heel/lance squeeze, personalized interventions

## Abstract

**Background:** Assessing pain in infants is challenging due to their inability to communicate discomfort. Accurate pain evaluation is essential, as unaddressed pain might lead to long-term neurological consequences. This study investigates the use of conventional two-site near-infrared spectroscopy (NIRS) to evaluate hemodynamic responses in the prefrontal cortex during nociceptive stimuli. **Methods:** Data were prospectively collected from ten infants undergoing elective heel lance/squeeze (HLS) after surgery. Continuous bilateral NIRS oxygenation monitoring was performed alongside cardiorespiratory and behavioral (Children’s and Infant’s Postoperative Pain Score (CHIPPS)) pain assessments before, during, and after HLS. The primary outcome was the correlation between NIRS response and CHIPPS. **Results:** The average gestational and postnatal ages were 39 weeks and 49 days. No significant changes in prefrontal oxygenation levels (left, right, combined, ipsilateral, contralateral) were observed during the first ten seconds of HLS compared with baseline. Although CHIPPS and heart rates increased, oxygenation levels remained unchanged throughout the entire HLS event. Significant fluctuations in oxygenation levels from baseline were recorded across all optode configurations, with changes in the lowest oxygenation levels at the contralateral and left hemispheres inversely correlated with CHIPPS and HR changes. **Conclusions:** While there were subtle alterations in NIRS signals suggesting potential nociceptive-evoked changes, these were inconclusive. By design, the utilized two-site conventional NIRS system may not effectively detect acute pain. Future studies on prefrontal cortical pain processing could benefit from confirmatory NIRS signals from the primary somatosensory and motor regions. Integrating data from fNIRS, fMRI, EEG, along with sympathetic indicators like skin conductance and heart rate variability, would improve the quantification of cortical pain processing in non-verbal infants.

## 1. Introduction

Hospitalized infants and neonates often undergo painful experiences. Research has linked pain to short-term cardiovascular and respiratory effects [1,2], as well as long-term neurological consequences, including changes in brain structure, behavior, cognitive, and motor development [3,4,5]. Additionally, altered pain thresholds have been documented in school-aged children [6,7,8,9] and even young adults [10] who have experienced pain in infancy. Due to the nonverbal nature of infants’ responses to pain, various observational tools have been used as potential pain indicators, focusing on behavioral aspects (such as autonomic activity, grimacing, crying, and motor activity), physiological responses (like cardiorespiratory parameters), or a combination of both. However, none of these unidimensional or multidimensional pain measures have been established as the ‘gold standard’ for detecting acute pain in nonverbal infants [11]. In adults, self-reported pain experiences are considered the ‘gold standard’ and correlate well with cortical activity induced by pain [12]. The cerebral cortex plays a crucial role in modulating nociceptive inputs and processing these inputs into subjective pain experiences [13]. Neurophysiological measures may offer close estimates of pain detection at the cortical level. Certain cerebral activities evoked by nociception have been studied in neonates [14] and may be influenced by analgesics [15]. As such, non-invasive brain imaging could serve as a potential pain indicator as well as a measure of analgesic efficacy [14]. Neurophysiological imaging techniques, including electroencephalography (EEG), functional magnetic resonance imaging (fMRI), and near-infrared spectroscopy (NIRS), have been increasingly applied to the assessment of acute pain in infants. Unlike NIRS and fMRI, which provide high accuracy in measuring hemodynamic changes, EEG directly records pain-related neuronal electrical activity. However, while EEG has shown methodological consistency, it suffers from poor spatial resolution and has not been widely applied [11]. NIRS measures regional tissue oxygenation by utilizing the differential absorption spectra of near-infrared light, which is affected by oxygenated and deoxygenated hemoglobin. This technique visualizes real-time changes in concentrations of oxy- and deoxyhemoglobin in capillary networks close to activated neural circuits. Based on the assumption that neuronal activity is closely related to hemodynamic changes, cerebral oxygenation measured by NIRS can indicate the brain’s response to nociceptive stimuli. However, these oxygenation changes are also influenced by various factors, such as medications, severity of illness, fluctuations in blood pressure, changes in oxygen and carbon dioxide levels, acid–base status, and immaturity [16]. Nociceptive stimuli—unlike non-nociceptive control stimuli—have been shown to increase cerebral oxygenation over the somatosensory cortex in infants and preterm neonates, highlighting the potential for higher, cortical-level pain processing information from 25 weeks on [17,18]. Heel lance/squeeze (HLS) procedures have been employed to characterize nociceptive-associated hemodynamic changes across somatosensory and other cortical areas using NIRS [19,20]. Additionally, NIRS has been used to quantify the effectiveness of oral sweet solutions for pain relief [21]. Functional MRI studies indicate that both somatosensory and prefrontal regions can respond to nociceptive stimuli. By using blood-oxygen-level-dependent (BOLD) fMRI, researchers have detected an integrative network of brain regions crucial for processing sensory input and behavioral responses to pain, known as the descending pain modulatory system. This includes the primary somatosensory cortex, the prefrontal cortex, the anterior cingulate cortex, the thalamus, the insula, and the amygdala, observed in both adults and infants. More specifically, the frontal cortices and anterior cingulate orchestrate top-down influences on the periaqueductal grey and posterior thalamus and thereby gate pain modulation [22]. Notably, activation in the prefrontal cortex—specifically, the middle frontal gyri within the dorsolateral prefrontal cortex—has been identified in fMRI studies where an infant’s foot was subjected to mechanical nociceptive stimulation [23,24,25]. These fMRI studies that show BOLD changes, particularly in the prefrontal cortex, have also been corroborated by multichannel functional NIRS (fNIRS) studies [26,27,28,29,30]. Given these findings, this study aimed to quantify the capability of bifrontal NIRS to reflect cerebral hemodynamic processing related to the cortical perception of nociceptive stimuli in infants undergoing postoperative care. In light of the widespread use of bifrontal-placed NIRS for hemodynamic monitoring in pediatric patients, especially the INVOS 5100C system, we speculated as to whether this device could detect hemodynamic frontal cortical alterations secondary to pain perception. The feasibility of detecting pain by this technique would be a clinically significant advancement in pain detection. While theoretical evidence supports the possibility of pain detection through NIRS, this feasibility study specifically focused on detecting bilateral NIRS signals at the forehead in response to nociceptive stimuli. Our results indicate no changes in oxygenation levels during the first ten seconds after HLS start and no changes during the entire HLS procedure compared with pre-stimulus levels. Moreover, distinct NIRS signal alterations that may potentially reflect nociceptive-evoked hemodynamic changes in the frontal region might have been too unspecific to draw definite conclusions.

## 2. Patients and Methods

**Figure 1:**
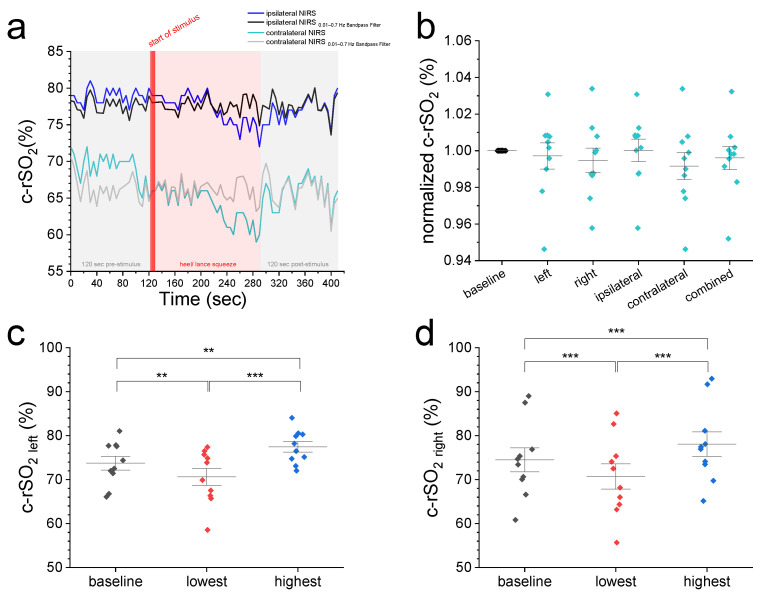

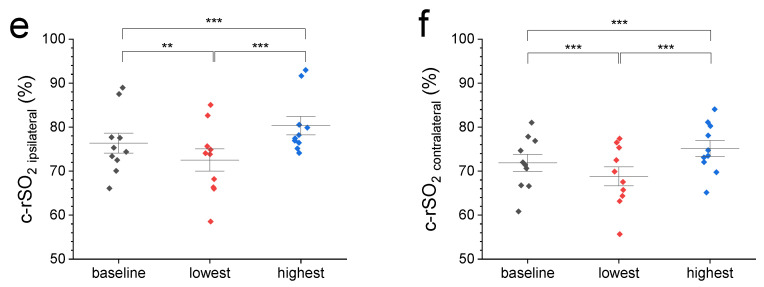
(**a**) A representative c-rSO_2_ trace illustrating a contralateral-sided decrease in oxygenation in the prefrontal cortex upon a nociceptive HLS stimulus before (green trace) but not after Bandpass filtering for artifact removal (grey trace). (**b**) Comparison of normalized 2 min baseline c-rSO_2_ levels with normalized c-rSO_2_ averages during first 10 s after nociceptive stimulus with no differences among NIRS configurations (*p* value range: 0.51–1). (**c**–**f**) Comparison of 2 min baseline c-rSO_2_ levels with the lowest and highest c-rSO_2_ levels during an HLS event at various NIRS configurations (left, right, ipsilateral, and contralateral hemisphere). Data are expressed as mean ± SEM. ** (*p* ≤ 0.01), or *** (*p* ≤ 0.001).

This prospective feasibility study involved ten infants who underwent continuous postoperative cerebral NIRS monitoring during nociceptive heel lance/squeeze (HLS) stimuli after open inguinal hernia repair at a single tertiary pediatric surgery center in Germany from January to June 2021. Eligible infants were under 12 months, without prior surgeries or painful experiences. Exclusion criteria included age over 12 months, poor NIRS or vital parameter signal quality, lack of parental consent, and medical conditions such as metabolic, pulmonary, renal, cerebral, or cardiac diseases, hypoxic-ischemic encephalopathy, previous intraventricular hemorrhage or periventricular leukomalacia. Out of the 14 infants initially recruited, 2 were excluded due to poor NIRS signal quality, and 1 was excluded due to a previous painful experience that could not be ruled out. Parental consent was withdrawn for another infant after inclusion. The primary objective was to correlate the Children’s and Infant’s Postoperative Pain Scale (CHIPPS) score with prefrontal cortical NIRS during nociceptive HLS stimuli. Secondary objectives included assessing CHIPPS scores, cerebral NIRS, heart rate (HR), breathing rate (BR), and peripheral oxygen saturation (SpO_2_) before, during, and after the HLS stimuli, as well as spatial differences in NIRS response to unilateral stimuli. All infants received awake caudal anesthesia for surgery, and analgesics were given at the discretion of the anesthesiologist or nurse. The CHIPPS score, validated for assessing postoperative pain in children aged 3 months to 5 years, encompasses five items—crying, facial expression, trunk posture, leg posture, and motor restlessness—each rated from 0 to 2. The total score ranges from 0 (no pain) to 10 (maximum pain) [31]. Assessments were conducted at 15 s intervals, with scores of 4 or higher indicating the need for analgesic therapy. Cerebral regional oxygenation (c-rSO_2_) is a parameter derived from NIRS technology that is based on the principle of differently absorbed spectra of oxygenated and deoxygenated hemoglobin. Emitted near-infrared light at two wavelengths (730 and 810 nm) penetrates the underlying tissue to a depth of 2–3 cm, with reflected light detected by two photodetectors (optodes) placed 3 and 4 cm from the light-emitting diode. A device-specific algorithm calculates and displays tissue oxygenation as the percentage of oxygen-saturated hemoglobin relative to total hemoglobin. An INVOS 5100C^®^ device (Covidien, Mansfield, MA, USA) was used with bilateral placement of optodes (OxyAlert Neonatal NIRSensor^®^, Covidien) on the forehead 1 cm over the eyebrow, as represented in Figure S1, covering the Fp1 and Fp2 corresponding positions of the international 10–20 electroencephalogram electrode placement system. To assess cortical responses to nociceptive events, the HLS location was documented, and NIRS signals were analyzed offline. NIRS raw data were collected using the INVOS^®^ analytics tool (Version 1.2.1, Somanetics Corporation, Troy, MI, USA) at an internally set sampling frequency of 0.16 to 0.2 Hz and were later converted to Excel^®^ (Microsoft, Inc., Redmond, WA, USA). An optical check of raw data quality was performed in each case after the data underwent bandpass filtering at 0.01 and 0.7 Hz to minimize artifacts. To improve interindividual comparability, data underwent further normalization by individually dividing mean data at T2 and T3 (Table 1) and the first ten seconds after HLS (Figure 1b) by the corresponding two-minute baseline mean rSO_2_ data from T1. The SpO_2_ (%), HR (beats per minute), and BR (breaths per minute) were continuously measured using a Philips IntelliVue X2^®^ patient monitor (Philips, Amsterdam, The Netherlands). NIRS monitoring commenced once the infant had reached stable cardiorespiratory status following transfer from the recovery room to the ward. The HLS procedure was part of the routine blood gas analysis (BGA) sampling, and no patient underwent HLS solely for the study’s purpose. HLS was administered when (1) motor block was diminished, as evidenced by spontaneous movement in the lower extremities; (2) a gentle pinch on the intended HLS site confirmed reduced caudal anesthesia 15 to 20 min before the stimulus; (3) based on literature data [32], the HLS was performed at least four hours after caudal block initiation (with Bupivacaine^®^) to ensure restored pain sensation; and (4) to minimize confounding factors from surgical site pain, HLS was only performed when the infants were in a calm, resting state, with no indications of pain for at least 15 min. The pre-selected HLS site was cleansed with a 70% alcohol swab. Following the HLS, an automatic Unistik^®^ 3 Neonatal device (18G; 1.8 mm penetration depth; Owen Mumford GmbH, Großostheim, Germany) was used at a prewarmed lateral heel of one selected extremity, and the site was squeezed for a variable duration until the capillary was adequately filled for BGA analysis. All patients were placed in a supine position with the head kept midline at 0°. Moreover, to reduce movement artifacts and their effects on circulation during measurements, the head and body positions were kept unchanged during HLS. NIRS measurements were taken every five to six seconds, while BR, HR, and SpO_2_ were recorded every second. The CHIPPS was assessed every 15 s, following the protocol proposed by Büttner et al. [31]. Averages were calculated for 120 s before (T1; baseline) and after (T3) an HLS event of variable duration (T2; 3.5 ± 2.2 min). Accounting for the initial nociceptive heel lance stimulus, the first ten seconds following the HLS were analyzed and compared with pre-stimulus values at T1.

Statistical analysis was conducted using OriginPro 2021^®^ (OriginLab, Northampton, MA, USA). For parametric data, means and standard deviations (SD) were reported; non-parametric data were summarized using medians and 1st and 3rd quartiles (Q1–Q3). Normal distribution was confirmed with the Kolmogorov–Smirnov test at a significance level of 0.05. Parametric data were compared using repeated measures analysis of variance (ANOVA) with Tukey’s multiple comparisons post hoc test. Adjustments for violations of Mauchly’s test of sphericity were made using the Greenhouse–Geisser or Huynh–Feldt corrections. Non-parametric data were compared using Friedman ANOVA with Dunn’s post hoc test. The Kendall rank correlation coefficient (Kendall’s Τ) was employed to evaluate the relationship between ΔCHIPPS and the various Δc-rSO_2_ constellations (combined bilateral average, contralateral, ipsilateral, left hemisphere, right hemisphere). The difference between the highest and lowest c-rSO_2_ values during HLS and the 2 min pre-stimulus baseline average was taken as Δc-rSO_2_. A *p*-value of ≤0.05 was regarded as statistically significant, with differences noted as * (*p* ≤ 0.05), ** (*p* ≤ 0.01), or *** (*p* ≤ 0.001). Sample size calculations were performed using G*Power 3.1^®^ [33]. The existing literature does not provide comparable data on the correlation between CHIPPS and cerebral NIRS. Kumar et al. [34] found a positive correlation between cerebral oxygenation changes and two behavioral measures in preterm infants during HLS events: the Neonatal Infant Pain Scale (NIPS; r = 0.71) and the Premature Infant Pain Profile—Revised (PIPP-R; r = 0.78). With 80% power, a 0.05 alpha level, and a 10% attrition rate, the estimated minimum sample sizes for correlating c-rSO_2_ with CHIPPS were 8 to 10. This study received approval from the Ethics Committee of Ruhr University, Bochum (registration no. 19-6738-§ 23b) and adhered to the principles outlined in the Declaration of Helsinki. Written informed consent from parents was obtained before the commencement of measurements.

## 3. Results

A total of ten male infants underwent bilateral frontal cerebral NIRS monitoring during elective postoperative HLS. The mean ( ± SD) gestational age was 39 ± 2 weeks, while the postnatal age and weight at the time of surgery were 49 ± 23 days and 5.0 ± 0.9 kg, respectively. The blood gas values were within normal ranges. The duration of surgery was 37 ± 13 min, and the anesthesia duration, from induction to transfer to the ward, was 75 (65–90) min. When comparing the mean raw and normalized c-rSO_2_ data across various configurations—including left, right, combined (bilateral average), ipsilateral, and contralateral hemisphere—no significant differences were observed during the HLS (T2) or after the procedure (T3) when compared with baseline values at T1 (see Table 1).

We then compared the 2 min pre-stimulus baseline c-rSO_2_ levels from different configurations with normalized values from the first 10 s after the start of nociceptive heel lance stimulus (Figure 1a,b) and the highest and lowest observed levels during HLS (Figure 1c–f). Figure 1a displays representative ipsi- and contralateral c-rSO_2_ raw data before and after Bandpass filtering. Comparison of normalized 2 min baseline c-rSO_2_ levels with normalized c-rSO_2_ averages during first 10 s after stimulus (Figure 1b) showed no differences among the NIRS configurations (*p*-value range: 0.51–1). Compared with pre-stimulus baseline values, both the highest and lowest c-rSO_2_ levels during HLS at all configurations displayed a significant increase/decrease from baseline oxygenation levels (Figure 1c–f).

**Figure 2:**
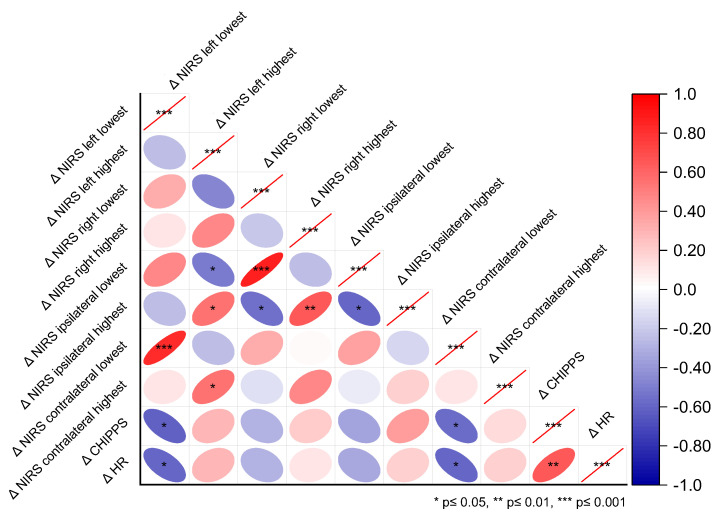
Correlations between the highest and lowest c-rSO_2_ changes from baseline (Δc-rSO_2_) and the reference pain measures ΔHR and ΔCHIPPS during nociceptive heel lance/squeeze procedures, considering the different NIRS optode configurations—contralateral, ipsilateral, left, and right hemispheres.

We conducted a correlation analysis between the changes (Δ) of the highest and lowest c-rSO_2_ levels during the HLS from the pre-stimulus baseline and the changes in the reference measures HF and CHIPPS. The lowest Δc-rSO_2_ levels from both the contralateral and left hemispheres during HLS were inversely correlated with ΔCHIPPS (contralateral r = −0.57, *p* = 0.024; left r = −0.61, *p* = 0.015; see Figure 2) and ΔHR (left and contralateral r = −0.60, *p* = 0.016). Moreover, ΔCHIPPS was positively correlated with ΔHR (r = 0.66, *p* = 0.009).

## 4. Discussion

In this pilot study, we investigated the feasibility of assessing bilateral prefrontal cortical hemodynamic responses to nociceptive stimuli in a cohort of infants who underwent elective inguinal herniotomy. Although the nociceptive stimuli used were considered painful—evidenced by elevated CHIPPS and HR—neither the normalized ten-second mean oxygenation values accounting for nociceptive stimulus nor the mean oxygenation levels during the entire HLS procedure were altered. However, when we compared changes in the highest and lowest c-rSO_2_ values during HLS with the pre-stimulus baseline, we observed alterations in c-rSO_2_ levels across all NIRS optode configurations. These findings are consistent with other studies that have reported decreased oxygenation levels during cortical activation that reflect different states of oxygen demand. Further, the lowest Δc-rSO_2_ levels from both the contralateral and left hemispheres during HLS were inversely correlated with ΔCHIPPS and ΔHR.

Previous neuroimaging studies suggest that the cortical processing of pain induces hemodynamic responses within the prefrontal cortex measurable by fMRI and fNIRS [22,23,24,25,26,27,28,29,30]. Generally, activities in the prefrontal cortex may relate to the evaluation and anticipation of pain and the emotional regulation of stress evoked by painful stimuli [35]. Chronic pain has been associated with reduced gray matter density in the dorsolateral prefrontal cortex compared with controls [35]. Additionally, the prefrontal cortex indirectly modulates stress through its regulation of the hypothalamus, which controls the hypothalamic–pituitary–adrenal axis and sympathoadrenal activity, thereby orchestrating the stress response [36,37]. Currently, knowledge regarding pain processing in infants remains limited. Furthermore, as the maturation of affective and cognitive circuits is not fully complete at birth, pain experiences may differ across pediatric cohorts, potentially explaining differences in cortical pain processing between children and adults [22]. The continuous, non-invasive NIRS technique for hemodynamic assessment of cerebral metabolism and perfusion has gained wide acceptance. It has become the standard of care for monitoring neonates and critically ill infants in the neonatal intensive care unit (NICU) and during anesthetic procedures by providing real-time information on cerebral oxygenation [38,39,40]. However, this information should be interpreted in the context of other physiological parameters. The application of conventional NIRS optodes to the forehead presents a promising method for assessing the cortical hemodynamic response to pain. The frontal NIRS application has the potential to overcome technical and transparency issues that restrict the use of somatosensory cortex NIRS signals—proven as effective pain surrogates [18]—due to hair coverage over parietotemporal sites. Additionally, NIRS optodes placed for other purposes may also serve as pain estimators, potentially offering relative ease in signal gathering and interpretation compared with more complex techniques such as EEG, fNIRS, and fMRI, the latter of which presents significant logistical challenges. One concern is that, if pain induces measurable hemodynamic changes in the cortex, this may confound the hemodynamic monitoring intended for the assessment of cerebral oxygenation status rather than pain detection. Literature on conventional one- or two-optode frontal NIRS measurements for pain assessment is limited and inconsistent, with conflicting results stemming from a large variety of methodological approaches and investigated cohorts. Table 2 summarizes data from 13 studies involving 917 patients (718 children [32,34,41,42,43,44,45,46,47] and 199 adults [48,49,50] that utilized frontal (forehead) NIRS optode placements with five different devices for assessing pain processed in the frontal cortex. Despite three studies focusing on adults—the population for which independent self-reporting serves as a ‘gold standard’—results have been contradictory. Gelinas et al. found divergent outcomes in oxygenation levels during bilateral hemodynamic monitoring in patients post-cardiac surgery: one study reported a bilateral decrease [49], while another indicated a bilateral increase [48] upon painful stimuli. One possible explanation from the authors could be the circumstances under which the nociceptive stimulus was applied. In their first study, c-rSO_2_ increased in response to the insertion of intravenous and arterial lines. However, in their second study, during the removal of mediastinal tubes, c-rSO_2_ decreased. According to the authors, this decrease may have resulted from an increased cerebral venous return due to elevated intrathoracic pressure and reduced cerebral venous outflow. This change could be linked to the Valsalva-type reaction that occurs when holding one’s breath during tube removal. Interestingly, Mukaihara et al. found an increase in oxygenation in the contralateral prefrontal cortex of adults when subjected to painful stimuli during thoracotomy, most likely not addressed to a Valsalva-type reaction [50]. In pediatric studies, decreased frontal oxygenation levels were observed in seven studies [34,41,42,43,45,46,51], one study indicated increased levels [44], and three other studies found no changes in NIRS signals during nociceptive stimuli [42,46,47]. The latter findings align with our results and are noteworthy as all three studies used only a single optode on the left forehead [47] or did not specify the forehead position [42,46]. Data on frontal NIRS regarding the affected side and whether the oxygenation increases or decreases in response to nociceptive stimuli remain conflicting. We therefore considered both the highest and lowest changes from baseline during nociceptive stimuli (HLS) for analysis and correlated these changes with referent pain measures such as CHIPPS and HR. Distinguishing between sides of the prefrontal cortex (left vs. right) differs from analyzing stimulus-adjusted data, as in the discrimination between ipsilateral and contralateral cortices concerning a one-sided stimulus. This distinction is important as studies have demonstrated sidedness in cortical response, with observed frontal ipsilateral and contralateral activation. For instance, Sakuma et al. found that Oxy-Hb levels in the contralateral prefrontal cortex decreased during pain stimulation in the gingiva using fNIRS [28]. Most studies referenced in Table 2 focused primarily on alterations without thoroughly examining the cortical side in relation to the nociceptive stimulus. Among the studies utilizing bilateral NIRS [32,44,48,49,50], only Ozedmir and Mukaihara [44,50] analyzed stimulus-dependent ipsi- and contralateral NIRS signals. Among the eight studies that employed one-sided NIRS optode placements, three used central placement [41,45,51], three did not specify placement [42,43,46], and one used contralateral [34] or left-sided placement [47] in relation to the nociceptive stimulus. These methodological choices may have led to overlooked data from the opposite side, contributing to discrepancies in results that make direct comparisons of findings challenging. For example, utilizing only one optode means a potential signal on the opposite side could go undetected. Further, the central placement of the optode may lead to confounding results due to the frontal sinus, which can reduce the NIRS signal sensitivity [52,53]. Moreover, via central optode placement, the cortical NIRS signal may interfere with that form of the superior sagittal sinus, increasing the variability and thus not accurately reflecting brain tissue hemodynamics [35]. Frontal asymmetry (FA) can only be observed using at least two optodes or a multielectrode fNIRS system. An asymmetry in prefrontal activation may be linked to mental stress, as demonstrated by EEG and NIRS studies showing that infants exhibit greater relative right frontal activation compared with the left when processing negative or withdrawal emotions [54,55,56]. In our study, we noted distinct differences in oxygenation levels between both sides of the brain, suggesting that FA cannot be ruled out in our patient group. In contrast to these findings, Ozawa et al. [32] reported that painful stimulation evoked bilateral frontal cortical activation in full-term infants, regardless of prior experience with painful procedures. However, in preterm infants who had undergone numerous skin-breaking procedures, the response to pain stimuli resulted in greater left than right prefrontal cortical activation. This greater left-sided response partly aligns with our observations: While we did not find any oxygenation alterations in the ten-second HLS analysis (Figure 1b) and also during the entire HLS analysis (Table 1) when compared with baseline levels, the correlation analysis (Figure 2) revealed an inverse relationship between the lowest Δc-rSO_2_ levels from the left and contralateral hemisphere during HLS and changes in ΔCHIPPS and ΔHR. Whether these findings in term infants relate to their surgical history, similar to the preterm infants in Ozawa’s study, remains uncertain due to the limited data available on postsurgical pain processing in this age group. However, the short interval between surgery and pain measurement in our study makes it unlikely that structural connectivity changes in cortical circuits have occurred, explaining the observed asymmetric frontal response.

Interestingly, Missana et al. [58] investigated the processing of facial expressions of pain and anger in 8-month-old infants and adults, utilizing event-related brain potentials alongside frontal EEG alpha asymmetry. They found opposite patterns in FA responses to pain and anger between infants and adults, suggesting that developmental and individual differences in the neural processing of dynamic expressions of pain and anger may exist. Although our correlation findings on oxygenation changes may indicate a specific pattern of prefrontal activation associated with pain, reminiscent of FA, our HLS event analysis on cerebral oxygenation during the first ten seconds and also during the entire event showed no significant differences when compared with the baseline before HLS, aligning more closely with findings from Dewi et al. [42]. They also reported no differences during automatic HLS, although the site of their single electrode was not specified. However, despite showing behavioral reactivity, some infants do not exhibit an EEG or NIRS response to nociceptive or innocuous somatosensory stimuli. This lack of response may indicate individual variability in pain sensitivity, which may be influenced by genetic and epigenetic factors [59,60,61], consistent with the variability in facial expressions following HLS in preterm and term infants [7]. In the study by Hwang [51], who used one central optode, the cerebral oxygenation decreased during and after a manual HLS in preterm infants, but not in the automatic HLS group. This may indicate an insufficient pain stimulus in their study regarding the automatic HLS. Similarly, the utilized automatic lancet may have caused an insufficient nociceptive stimulus in our patients. This insufficient stimulation could also relate to our cases, particularly if the caudal block at the lower extremity partly persisted even though we only performed HLS after confirming that nociceptive and motor functions had been restored, which typically occurred 3 to 6 h [32] after the administration of caudal block. Habitual effects might explain the responses observed in Hwang’s group [51] of premature patients, who likely had experienced significant pain before. In their study, only a more intense pain stimulus, as presumed to be the case with non-automatic lancet types, could evoke nociceptive cortical responses detectable by NIRS. Recommended behavioral scores have been used as controls for NIRS, with the Premature Infant Pain Profile (PIPP) or its revised form, the PIPP-R, being the most utilized, followed by the Neonatal Infant Pain Scale (NIPS) and other self-reports. Most studies found correlations between behavioral measures and NIRS levels [32,43,49] or changes from baseline levels [34], while others found no correlations [32,44]. In this context, Kumar et al. [34] found a positive correlation between cerebral oxygenation changes and the NIPS, with r = 0.71, and with the PIPP-R, at r = 0.78, in preterm infants during HLS events, where the NIRS signal was derived from the forehead on the contralateral side to the prick. Similar to Kumar et al.’s experimental design correlating NIPS and PIPP-R with changes in c-rSO_2_ from baseline during HLS, we also found that the changes in the CHIPPS and Δc-rSO_2_ were correlated, thereby contributing data from another behavioral pain measure. Additionally, the periods for baseline assessment of pre-stimulus and the times for averaging levels during and after stimulation are inconsistent among various studies. When considering 2 min means before and after the HLS, we did not find altered cortical NIRS levels (Table 1). However, when evaluating changes in rSO_2_ by analyzing the highest and lowest levels during the stimulus from a defined 2 min pre-stimulus baseline—as undertaken by Kumar [57] and Devi et al. [42], who calculated changes in rSO_2_ (ΔrSO_2_) using the baseline and the minimum observed rSO_2_ during the 30 s after the prick—we did find differences. Nonetheless, the duration of the relevant hemodynamic response that indicates a real painful experience remains unknown.

### Limitations

This study has several limitations. Although we did not include infants who underwent prior nociceptive stimuli or surgery, all infants participating in the study had surgery a few hours before measurements began, potentially resulting in pain and cortical processing before the start of our assessments. As prior pain is known to influence pain response in both term and preterm infants, the inclusion of infants who experienced nociceptive stimuli during surgery may have affected their pain thresholds. Prior pain may disrupt the relationship between cortical and behavioral measures of pain, as evidenced by data from Ozawa et al. [32], indicating that prior experiences of pain should be taken into account when assessing pain. Slater et al. [7] found that nociceptive-evoked EEG potentials following HLS were larger in ex-premature infants compared with age-matched term-born infants, likely due to their previous experiences of pain. Prior pain could also impact behavioral pain measure scores in both preterm [62] and term-born infants [63], with studies suggesting that this effect may persist into childhood and adulthood [64,65]. In this regard, we only performed the HLS event when children were calm, as indicated by a CHIPPS score of less than 4 and a normal heart rate. However, undetected pain on one side may have affected the results, especially in the frontal near-infrared spectroscopy (NIRS) measurements. It is possible that the cortical nociceptive-induced hemodynamic responses were obscured by an activated frontal lobe due to the processing of pain, either from a permanent underlying pain at subthreshold levels or from acute pain at the surgical site.

Pain-related hyperventilation causing hypocapnia may be another potential confounding factor for cerebral oxygenation [28]. Carbon dioxide (CO_2_) is a known vasodilator, so a decrease in end-tidal CO_2_ may cause global cerebral vasoconstriction, potentially altering NIRS signals that might mimic cortical responses to pain. Unfortunately, we did not measure CO_2_ levels during HLS, but the CO_2_ levels from blood gas analysis were within normal values.

Although our sample size met the a priori calculations for the primary outcome, the small number of infants may have limited the ability to differentiate pain-related subtle variations from physiological or instrumental fluctuations. Additionally, our study was conducted exclusively with male infants. Given the known sex-dependent differences in pain-related brain activity in female infants—such as more intense and widespread spatial distribution of pain, as measured by EEG [66] —our findings might have differed in a more balanced cohort. For instance, females are reported to express pain through more pronounced facial expressions [65] and higher-pitched crying [66] compared with males. As a result, if females had been included, we might have detected more distinct pain-evoked variations in NIRS and CHIPPS.

By bandpass filtering our NIRS raw data, we accounted for biasing noise from blinking and breathing, thus minimizing the likelihood of NIRS not reflecting the true pain response secondary to stimulus. In general, the observed alterations in the lowest and highest c-rSO_2_ levels from baseline during HLS (Figure 1c–f) and the thus-derived correlations (Figure 2) may also have been caused by unspecific and still unfiltered fluctuations. Such fluctuations may also reflect the incapability of the INVOS 5100C to detect distinct alterations in cerebral hemodynamic responses to pain. In this context, we do not consider the observed maximum and minimum changes from oxygenation levels to reflect cortical responses to pain. In addition, the device-specific fixed sampling frequency of 0.16–0.2 Hz might have limited the detection of faster-altering cortical pain-induced hemodynamic alterations.

Lastly, as we only examined bilateral prefrontal NIRS, we could not assess the relationship between somatosensory and prefrontal cortical activity. A combined assessment of both prefrontal and somatosensory activity by fNIRS is necessary to ensure redundancy, detect asymmetries, and contextualize the different signals related to perceived pain.

## 5. Conclusions

This feasibility study investigated the use of near-infrared spectroscopy (NIRS) to detect pain by monitoring bilateral signals at the forehead in response to nociceptive stimuli in infants. Theoretical evidence supports the possibility of detecting pain through NIRS. However, our results show no significant changes in prefrontal oxygenation levels during the first ten seconds after the onset of heel lance/squeeze (HLS) and throughout the entire procedure, when compared with baseline levels before stimulation. Although we observed subtle alterations in NIRS signals that could indicate nociceptive-evoked hemodynamic changes in the frontal region, these changes might also reflect instrumental limitations. Moreover, the sampling frequency of 0.16–2 Hz employed by the NIRS system may not be adequate to detect acute pain effectively. Therefore, conventional NIRS systems like the INVOS 5100C may not represent reliable tools for clinical pain detection. However, with advancements in technology, future studies on prefrontal cortical pain processing could benefit from examining confirmatory NIRS signals from the primary somatosensory (S1) and motor (M1) regions. Additionally, integrating data from functional NIRS (fNIRS), functional MRI (fMRI), and electroencephalography (EEG) studies, alongside sympathetic system indicators such as skin conductance measurements or heart rate variability assessments, would enhance the quantification of cortical pain processing in non-verbal infants.

## Figures and Tables

**Table 1:** Nociceptive events.

	Heel Lance/Squeeze Event			*p*		
	T1 Before	T2 During	T3 After	T1 vs. T2	T1 vs. T3	T2 vs. T3
CHIPPS	0 (0–2)	6 (4–8)	1 (0–5)	0.003	0.94	0.07
HR (1/min)	148 ± 6	167 ± 5	151 ± 8	0.023	0.91	0.053
BR (1/min)	33 (29–36)	30 (28–47)	41 (34–42)	1	0.45	0.61
SpO_2_ (%)	100 (99–100)	100 (99–100)	100 (100–100)	1	1	0.74
NIRS						
c-rSO_2 combined_ * (%)	74 ± 2	74 ± 2	74 ± 2	0.83	0.97	0.70
c-rSO_2 left_ (%)	74 ± 2	74 ± 2	74 ± 2	0.83	0.58	0.27
c-rSO_2 right_ (%)	75 ± 3	75 ± 3	75 ± 3	0.93	0.71	0.18
normalized c-rSO_2 combined_ *	1	1 ± 0.002	0.99 ± 0.002	0.90	0.89	0.66
normalized c-rSO_2 left_	1	1 ± 0.003	0.99 ± 0.002	0.87	0.52	0.26
normalized c-rSO_2 right_	1	1 ± 0.002	1 ± 0.003	0.35	0.99	0.99
adjusted to stimulus						
c-rSO_2 ipsilateral_ (%)	76 ± 2	77 ± 2	76 ± 2	0.44	0.99	0.36
c-rSO_2 contralateral_ (%)	72 ± 2	72 ± 2	72 ± 2	0.97	0.96	1
normalized c-rSO_2 ipsilateral_	1	1 ± 0.003	0.99 ± 0.002	0.48	0.96	0.34
normalized c-rSO_2 contralateral_	1	0.99 ± 0.002	0.99 ± 0.003	0.99	0.89	0.99

CHIPPS: Children’s and Infant’s Postoperative Pain Scale; HR: heart rate, BR: breathing rate; SpO_2_: peripheral oxygen saturation; c-rSO_2_: cerebral regional tissue oxygenation. Data presented as mean ± SD or median (Q1–Q3). * average of bilateral data.

**Table 2:** Synopsis of studies utilizing frontal cerebral NIRS for pain assessment.

Reference	Cohort	Procedure/Setting	NIRS-Device	Frontal Optodes (*n*)	Referent Behavioral Pain Measures	Cortical NIRS Outcome
This study	10 _t_	HLS after hernia repair	INVOS 5100C	2 (ipsi- vs. contralateral, left vs. right, and combined)	CHIPPS	No changes during first ten seconds and entire HLS event when compared with baseline c-rSO_2_ Inverse correlation of ΔHR and ΔCHIPPS with lowest Δc-rSO_2_ left and contralateral Rise in highest and lowest c-rSO_2_ levels during HLS from baseline in all configurations (unspecific?)
Sun et al. [41]	82 _p_	During ROP screening ^#^	EGOS-600A	1 (central)	PIPP-R	Decreased c-rSO_2_ in both groups (lower in control) during screening
Ozawa et al. [32]	80 _p&t_	During hand venipuncture ^##^	NIRO-200	2 (left vs. right)	PIPP	Terms w/o previous pain: facial expression PIPP-component correlated with bilateral activation (r_right_ = 0.53; r_left_ = 0.37; and total score) Terms with prior pain: no correlation between pain scores and cortical activation Prematures with prior pain: physiological PIPP component was correlated with bilateral activation (r_right_ = 0.36; r_left_ = 0.41)
Devi et al. [42]	180 _p_	Automatic vs. manual vs. needle HLS	INVOS 5100C	1 (forehead, n.s.)	PIPP-R	No difference in post-intervention c-rSO_2_ and Δc-rSO_2_ among groups; decreased c-rSO_2_ during HLS (*p*-values not stated); c-rSO_2_ normalization time higher with needle
Kumar et al. [57]	64 _p_	During HLS and venipuncture	INVOS 5100C	1 (contralateral)	NIPS, PIPP-R	Decreased c-rSO_2_ during both procedures; correlations between Δc-rSO_2_ and NIPS/PIPP-R at HLS (r = 0.71/r = 0.78) and at venipuncture (r = 0.66/r = 0.75)
Hwang et al. [51]	24 _p_	Manual vs. automatic HLS	INVOS 5100	1 (central)	PIPP	rSO_2_ decreased during and after manual HLS, but not in the automatic HLS group; rSO_2_ higher and c-FTOE lower after automatic HLS
Upadhyay et al. [43]	50 _p_	OG vs. NG tube insertion	INVOS 5100C	1 (forehead, n.s.)	PIPP-R	rSO_2_ decreased during tube insertion; lower rSO_2_ during NG insertion compared with OG; rSO_2_ normalization time higher with NG insertion; inverse correlation of rSO_2_ and PIPP-R (r = −0.20)
Ozdemir et al. [44]	119 _t_	HLS in LGA newborns vs. controls	Equanox 7600	2 (ipsi- vs. contralateral)	NIPS	Ipsi- and contralateral oxygenation increased after HLS in each group (no difference between groups); no correlation between NIRS and NIPS
Zhang et al. [45]	48 _p_	Pain during PICC	EGOS-600A	1 (central)	PIPP	rSO_2_ decreased during PICC
Kara et al. [46]	42 _p_	During ROP screening ^###^	INVOS 5100	1 (forehead, n.s.)	PIPP	No difference in c-rSO_2_ and c-FTOE before, during or after the exam among the groups; mild decrease in c-rSO_2_ within groups during ROP screening (*p*-values not stated)
Ranger et al. [47]	29 _p_	Calmer vs. facilitated tucking at HLS	Portalite Mini	1 (left)	BIIP	No differences between the groups in tissue saturation index during any of the epochs
Gélinas et al. [48]	40 _a_	Painful procedures ^####^	INVOS-4100	2 (left vs. right)	Self-report	Increased bilateral rSO_2_ during painful procedures; no association between ΔrSO_2_ and pain self-reporting
Gélinas et al. [49]	125 _a_	Non-painful vs. painful procedures ^####^	INVOS 5100	2 (left vs. right)	Self-report	Bilateral rSO_2_ decreases (<1%) during painful procedure; Critical-Care Pain Observation Tool score correlated with right rSO_2_ (r = 0.23)
Mukaihara et al. [50]	34 _a_	GA vs. GA and PVB at thoracotomy	NIRO-200	2 (ipsi- vs. contralateral)	n.a.	GA: ΔO_2_Hb (oxygenation) higher in the hemisphere contralateral to the side of surgery when the incision was made and 2 min after incision compared with ipsilateral side GA + PVB: no significant changes in the ΔO_2_Hb ΔtotalHb (blood volume) was higher in the contralateral hemisphere in the GA group at start of surgery

NIRS, near-infrared spectroscopy; HLS, heel lance/squeeze; ROP, retinopathy of prematurity; OG, orogastric; NG, nasogastric; LGA, large for gestational age (received more stimuli than controls); PICC, peripheral insertion of central catheter; GA, general anesthesia; PVB, paravertebral block; CHIPPS, Children’s and Infant’s Postoperative Pain Score; PIPP, Premature Infant Pain Profile; PIPP-R, Premature Infant Pain Profile—Revised; BIIP, behavioral indicators of infant pain; c-rSO_2,_ cerebral regional oxygenation; Δ, delta; HR, heart rate; c-FTOE, cerebral fractional tissue oxygen extraction; _p,_ prematures; _t_, terms; _a_, adults; ^#^, gentle human touch vs. control; ^##^, infants with vs. without previous pain; ^###,^ oral sucrose vs. intranasal fentanyl vs. intravenous fentanyl; ^####^, after cardiac surgery; n.s., not specified; n.a., not addressed.

## Data Availability

The raw data supporting the conclusions of this article will be made available by the corresponding author M.N., without undue reservation.

## Supplementary Materials

The following supporting information can be downloaded at https://www.mdpi.com/article/10.3390/jcm14072525/s1. Figure S1: Bilateral OxyAlert Neonatal NIRSensor^®^ optode placement with emitter (e, red), shallow (d1, yellow), and deep (d2, yellow) optode detectors and corresponding Fp1 and Fp2 (white circles) positions according to the international 10–20 electroencephalogram electrode placement system.

## Abbreviations

The following abbreviations are used in this manuscript:

CHIPPS	Children’s and Infant’s Postoperative Pain score
c-FTOE	Cerebral fractional tissue oxygen extraction
BOLD	Blood oxygenation level dependent fMRI
BGA	Blood gas analysis
BR	Breathing rate
FA	Frontal asymmetry
HR	Heart rate
NIRS	Near-infrared spectroscopy
HLS	Heel lance/squeeze
fMRI	Functional magnetic resonance imaging
fNIRS	Functional near-infrared spectroscopy
EEG	Electroencephalography
NIPS	Neonatal Infant Pain Scale
PIPP-R	Infant Pain Profile—Revised
ANOVA	Analysis of variance
ROP	Retinopathy of prematurity
c-rSO_2_	Cerebral regional oxygenation
SpO_2_	Peripheral oxygen saturation
