# Supplementary material for: Prefrontal Cortical Near-Infrared Spectroscopy for Acute Pain Assessment in Infants: A Feasibility Study
Source: J Clin Med. 2025 Apr 7;14(7):2525. doi: 10.3390/jcm14072525 (PMC11989543; doi:10.3390/jcm14072525)
Supplement: Supplementary file 1 [file jcm-14-02525-s001.zip › jcm-3477459-supplementary.pdf]

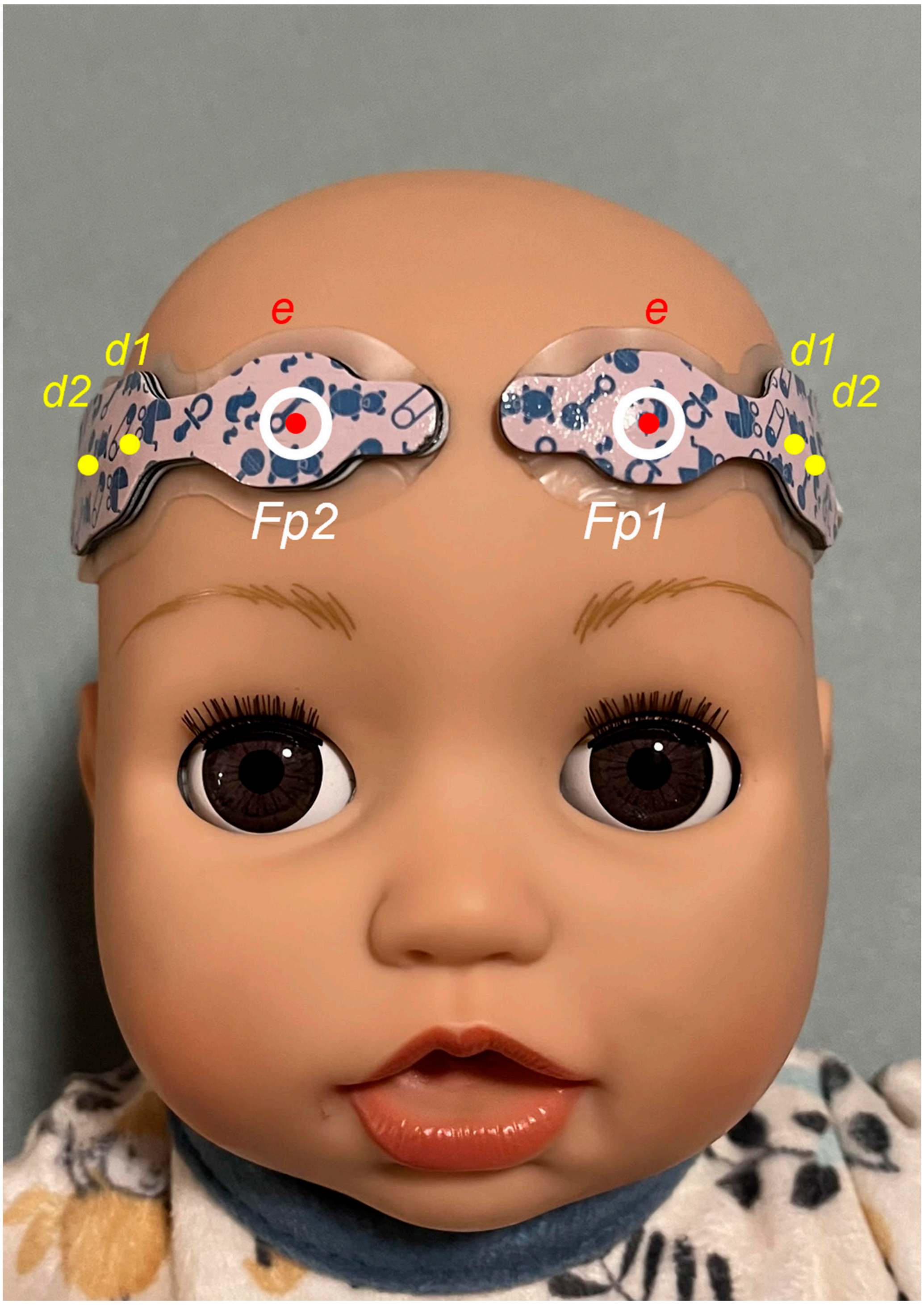

Figure S1. Bilateral OxyAlert Neonatal NIRSensor® optode placement with emitter (e, red), shallow (d1, yellow), and deep (d2, yellow) optode detectors and corresponding Fp1 and Fp2 (white circles) positions according to the international 10-20 electroencephalogram electrode placement system.
